# Donut and spheroid mitochondria: eating, regenerating or trash them out?

**DOI:** 10.1093/lifemeta/load008

**Published:** 2023-03-10

**Authors:** Wen-Xing Ding, Han-Ming Shen

**Affiliations:** Department of Pharmacology, Toxicology and Therapeutics, University of Kansas Medical Center, Kansas City, KS 66160, United States; Division of Gastroenterology, Hepatology and Mobility, Department of Internal Medicine, University of Kansas Medical Center, Kansas City, KS 66160, United States; Faculty of Health Sciences, Ministry of Education Frontiers Science Center for Precision Oncology, University of Macau, Macao 999078, China

**In response to stress, mitochondrion undergoes constant morphological changes, including the formation of donut and spheroid mitochondria, and both are believed to be implicated in its biological functions.**

Mitochondria are critical for cellular metabolic homeostasis and cell survival and death in eukaryotic cells. Mitochondria are dynamic organelles that constantly undergo fission and fusion. Mitochondrial homeostasis is tightly regulated by mitophagy for the removal of damaged or excess mitochondria and by mitochondria biogenies of new mitochondria. Mitochondria can also undergo other morphological transformation, such as formation of donut-like mitochondria or mitochondrial spheroids, and can also be secreted into the extracellular spaces. Here we discuss the mechanistic insights and physiological relevance of the donut-like mitochondria or mitochondrial spheroids and the secretion of mitochondria in cell biology.

**Figure 1:**
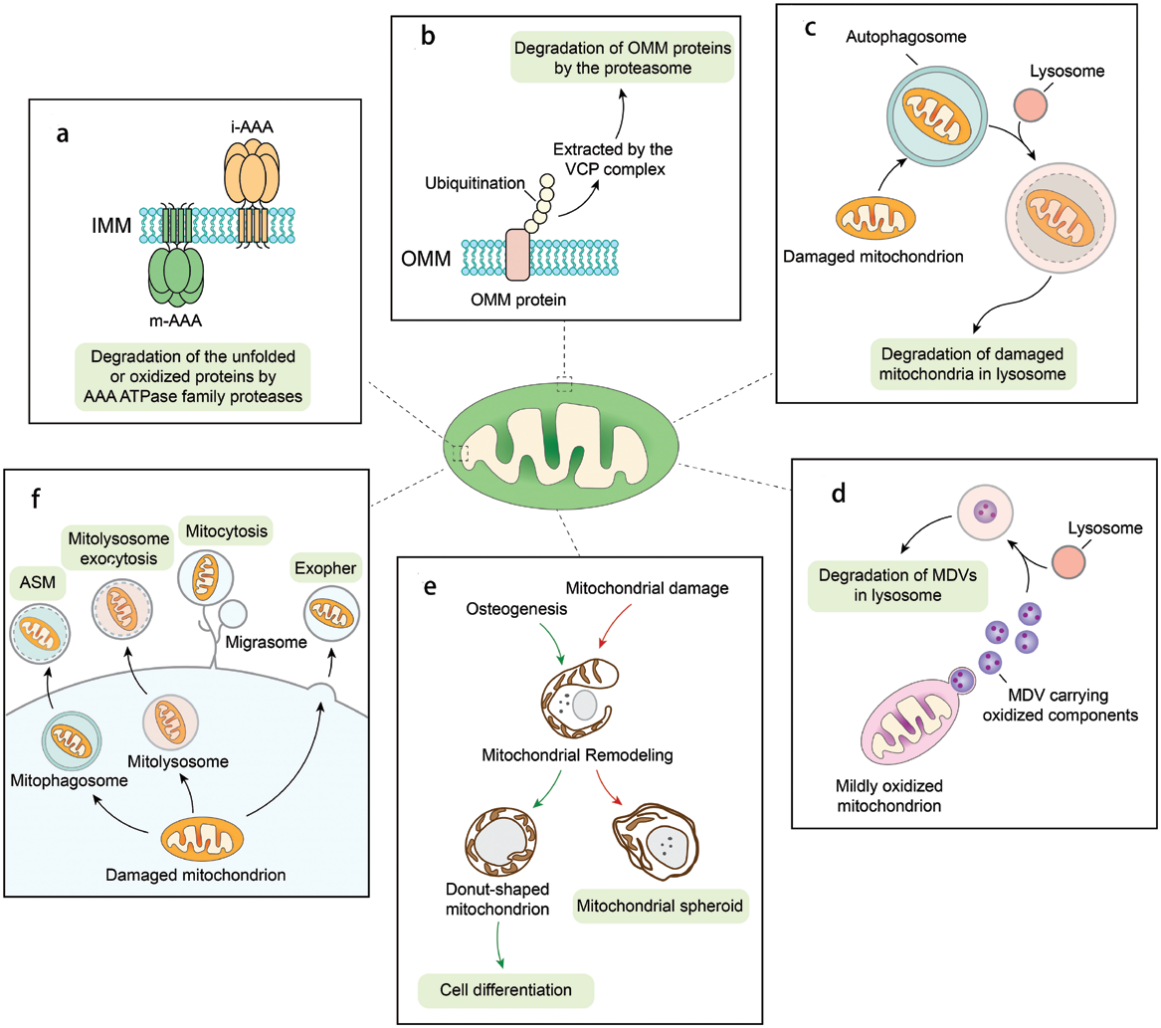
Diverse pathways in regulation of mitochondrial turnover. (a) Mitochondria have their own AAA ATPase family proteases located on both sides of the inner mitochondrial membrane (IMM), which are responsible for the degradation of the unfolded or oxidized proteins in the mitochondrial matrix and the intermembrane space. (b) Some outer mitochondrial membrane (OMM) proteins can be ubiquitinated and degraded by the ubiquitin-proteasome system. (c) Mitochondria are selectively cleared via selective autophagy, called mitophagy, in which the damaged mitochondria are enveloped by autophagosomes and trafficking to lysosomes for degradation. (d) A small portion of mitochondria can bud off to form mitochondria-derived vesicles (MDV) carrying oxidized mitochondrial proteins to lysosomes for degradation. (e) In response to a mitochondrial damage, depolarized mitochondria undergo dramatic morphological remodeling to form mitochondrial donuts or mitochondrial spheroids. (f) Mitochondria can be secreted out of cell as extracellular vesicles via processes such as mitocytosis, mitolysosome exocytosis, or autophagic secretion of mitochondria (ASM) as additional mechanisms to maintain mitochondria quality.

There are several well-studied mechanisms for mitochondrial turnover (Fig. 1). First, mitochondria have their own AAA ATPase family proteases located on both sides of the inner mitochondrial membrane (IMM), which are responsible for the degradation of the unfolded or oxidized proteins in the mitochondrial matrix and the intermembrane space [1]. Second, some outer mitochondrial membrane (OMM) proteins can be ubiquitinated and degraded by the ubiquitin-proteasome system, a process called mitochondria-associated degradation (MAD) [2]. Third, mitochondria are selectively cleared via selective autophagy, called mitophagy, in which the damaged mitochondria are enveloped by autophagosomes and trafficking to lysosomes for degradation [3, 4]. Fourth, a small portion of mitochondria can bud off to form mitochondria-derived vesicles (MDV) carrying oxidized mitochondrial proteins to lysosomes for degradation [5]. Fifth, in response to a mitochondrial uncoupler carbonyl cyanide *m*-chlorophenylhydrazone (CCCP) or a hepatoxic drug acetaminophen, depolarized mitochondria undergo dramatic morphological remodeling to form a ring- or donut-like structure, which was termed as “mitochondrial spheroid” [6]. Mitochondrial spheroid likely acts as an alternative mitochondrial quality control mechanism independent of autophagy-related 5 (ATG5) and ATG7 but is negatively regulated by Parkin, a Parkinson disease-related E3 ubiquitin ligase [6, 7]. Finally, mitochondria can secrete out of cell via extracellular vesicles, such as mitocytosis, mitolysosome exocytosis, or autophagic secretion of mitochondria (ASM) [8, 9]. While these complex mitochondrial quality control mechanisms have been well documented, most of these studies were conducted in cell culture system. The pathophysiological relevance of these pathways including MDV, mitochondrial spheroids, and secretion remains largely obscure.

In a recent study published in *Cell Metabolism*, Suh *et al*. [10] reported that fragmented mitochondria were donut-shaped and secreted out of cells during osteogenesis. To determine the role of mitochondrial changes in osteogenesis, Suh *et al*. used isolated osteoblasts (OBs) from genetically engineered mice that are *Col1a1-Cre* positive, which underwent differentiation with increased osteogenic markers during culture. Increased mitochondrial fragmentation was associated with decreased mitochondrial fusion protein optic atrophy 1 (OPA1) and increased mitochondria fission protein [phosphorylation of dynamin-related protein 1 (DRP1) at S616 and mitochondrial fission 1 protein (FIS1)] in cultured OBs. Careful morphological studies using the lattice structural illumination microscopy and transmission electron microscopy (TEM) revealed increased donut-shaped mitochondria and MDVs. Interestingly, increased membrane-surrounded mitochondria and MDVs were found in the culture medium of OBs, implying increased secretion of mitochondria and MDVs. Mechanistically, secretion of mitochondria and MDVs was markedly impaired in cluster of differentiation 38 (CD38)-deficient OBs but were significantly increased in cyclic ADP ribose (cADPR)-treated OBs. Functionally, secreted mitochondria and MDVs from matured OBs promoted osteogenesis *in vitro* and *in vivo*. Genetic deletion of OPA1 in OBs increased bone mass, whereas pharmacological activation of OPA1 impaired bone osteogenesis. Together, this study identified the unprecedented roles of mitochondrial morphological changes in maintaining mitochondrial homeostasis and in regulating cell development such as osteogenesis (Fig. 1).

While this study revealed an important physiological role of mitochondrial morphological transformation and secretion in osteogenesis and bone regeneration, many questions remain unanswered. First, the mechanisms of regulating the formation of donut-like mitochondria remains unclear. Since the number of donut mitochondria increased after knockdown of *Opa1* or overexpression of *Fis1*, one would assume that these donuts are fragmented mitochondria. However, these donuts may not necessarily be small based on the TEM images. The donut mitochondria increased in dynamin 1-like (*Dnm1l*) KO cells, and formation of mitochondrial spheroid is independent of DRP1, indicating that mitochondrial fission is dispensable for donut and spheroid mitochondria. Conversely, mitochondrial spheroids require mitofusin (MFN) 1 and MFN2, and are negatively regulated by Parkin [7]. The exact role of MFN1, MFN2, and Parkin for the donut mitochondria formation in cultured OBs was not carefully examined and remains unclear. Notably, Suh *et al*. took the circular/ring-like mitochondria under the fluorescence microscopy as the surrogate for the donut mitochondria under electron microscopy (EM). It remains uncertain whether the circular/ring-like mitochondria are really donut mitochondria under EM without clear correlative EM evidence. Second, the fate of these donut mitochondria deserves to be further studied. Suh *et al*. proposed that these donut mitochondria may promote MDV formation and secretion. This is somehow collaborative to a recent report in which damaged mitochondria caused by CCCP and mitochondrial respiratory chain complex inhibitors are cleared via extracellular release through a secretory autophagy pathway, a process defined as ASM [9]. Consistently, several other reports have demonstrated that the secretion of mitochondria might serve as a novel mode to maintain mitochondrial quality control, via processes such as mitolysosome exocytosis, mitocytosis, and exophers [8].

Mitochondrial spheroids obtain features of lysosomes and are likely degraded via lysosomes [7]. While increased mitophagy and mitochondria degradation were observed in cultured OBs, whether these donut mitochondria may end at lysosomes was not clear. Clearance of damaged mitochondria via lysosome independent of canonical autophagy machinery has been proposed and termed as micro-mitophagy. It would be of interest to examine the possibility that donut mitochondria or mitochondrial spheroid can be cleared via micro-mitophagy.

The functional implication of donut mitochondria or mitochondrial spheroid deserves further investigation. The study by Suh *et al*. revealed the role of donut mitochondria in maturation of OBs and osteogenesis via secretion. Notably, damaged mitochondria secreted via ASM are able to stimulate innate immunity with activation of the cyclic GMP-AMP synthase-stimulator of interferon genes (cGAS-STING) pathway [9]. It is of interest to further explore the functions of such mitochondrial morphological changes in other cellular or disease models.

In summary, the study by Suh *et al*., together with many others, has demonstrated donut-like mitochondria or mitochondrial spheroids as a unique form of mitochondrial morphology with specific functions. Further studies are needed to elucidate their exact physiological and pathological implications in health and disease.
